# Nationwide longitudinal population-based study on mortality in Italy by immigrant status

**DOI:** 10.1038/s41598-022-15290-8

**Published:** 2022-06-29

**Authors:** Anteo Di Napoli, Martina Ventura, Enrico Grande, Luisa Frova, Concetta Mirisola, Alessio Petrelli

**Affiliations:** 1 National Institute for Health, Migration and Poverty (INMP), Via di San Gallicano, 25a - 00153 Rome, Italy; 2 National Institute of Statistics (Istat), Rome, Italy

**Keywords:** Epidemiology, Risk factors

## Abstract

A systematic analysis of the mortality of immigrant residents throughout Italy has never been carried out. The present study aimed to evaluate differences in mortality by immigrant status. A longitudinal study of the Italian resident population (native and immigrants) recorded in the 2011 National Institute of Statistics Census was conducted. This cohort was followed up from 2012 to 2018 until death, emigration, or end of the study period. The exposure variable was the immigrant status, measured through citizenship, dichotomized into Italian and immigrant. The main outcome was overall and cause-specific mortality. Age-standardized mortality ratios (SMRs) were calculated. The SMRs among immigrants were half that of Italians, both for men (SMR 0.52) and women (SMR 0.51), with the lowest SMRs observed for subjects from North Africa and Oceania. For some causes of death, mortality was higher among immigrants: tuberculosis in both men (SMR 4.58) and women (SMR 4.72), and cervical cancer (SMR 1.58), complications of pregnancy, childbirth, and puerperium (SMR 1.36), and homicide (SMR 2.13) for women. A multivariable quasi-Poisson regression analysis, adjusted for age and macro area of residence in Italy, confirmed a lower all-cause mortality for immigrants compared to Italians, both for men (RR 0.46) and women (RR 0.44). Although immigration to Italy is no longer a recent phenomenon, and the presence of immigrants is acquiring structural characteristics, our study confirms their health advantage, with a lower mortality than that of Italians for almost all causes of death and for all areas of origin.

## Introduction

Many studies have found that immigrants generally have a lower all-cause mortality rate than natives, despite their lower socioeconomic status, a factor associated with poor health status, and their poorer living conditions^1–5^.

This issue represents an epidemiological paradox that has been typically explained in two ways.

The first explanation, the “healthy-migrant effect,” is based on the hypothesis that, due to the selective migration flows of healthy young people, migrants are healthier than the native people both of the country of origin and of the country of destination^1,2,6,7^.

The second is the so-called “salmon bias effect,” based on the hypothesis that older, unhealthy immigrants return to their country of origin for “the compulsion to die in one’s birthplace”^1,8–11^. In such a case, if deaths occurring in their country of origin are not registered in the mortality statistics of the country of residence, this may result in an artificially low immigrant mortality rate^1,7,10,11^. Furthermore, the fact that immigrants who returned to their country of origin remain in the demographic registers may determine an overcoverage of this population, meaning that the denominators are artificially inflate. This contributes to explaining the migrant mortality paradox, in particular at peak migration ages^13–15^.

Previous Italian studies found that immigrants showed a lower risk of mortality compared to Italians^2,16^. A recent study, which investigated the potential contribution of salmon bias effect in Italy, confirmed this finding; an underreporting of the deaths of immigrants who died abroad without an official residence change was observed. However, it was not enough to explain the large difference in mortality rates between immigrants and natives, probably confirming the relevance of the healthy migrant effect^2,13,15–18^.

Italy is a country with a short history of immigration, with a significant increase in the number of immigrants since the beginning of twenty-first century, in particular those coming from high migratory pressure countries. The number has doubled in the last 15 years, from 2.4 million people (4.1% of the resident population) in 2005 to 5.0 million (8.5%) in 2020^19^.

Few studies have been published on immigrant mortality in Italy^2,16,17,20,21^. To our knowledge, only two studies used a longitudinal approach^2,17^, but one of these was limited to the cohorts of residents of two cities, Turin and Reggio Emilia^2^.

A longitudinal approach in studies aimed at evaluating differences in mortality between immigrants and natives is useful because immigrants in host countries tend to lose their initial health advantage due to the ageing of the first cohorts, to the cultural and linguistic barriers in accessing health care, and to the acculturation process, leading immigrants to experience life conditions and behaviors similar to those of natives in the lower socioeconomic positions^2,22,23^.

As great variability in mortality among immigrants by ethnic group, area of residence in the host country, age, cause of death, and length of stay has been observed^22,24–27^, a study conducted on a nationwide basis may provide an exhaustive description of these factors.

A systematic analysis of the mortality of immigrants resident throughout Italy has never been carried out. The present study, based on the follow-up of the Italian population recorded in the 2011 Census, aimed to evaluate the differences in mortality by immigrant status in Italy.

## Methods

### Data sources, study population and design

The study adopted a longitudinal design in which the Italian population recorded in the 2011 Census was considered as the initial cohort. The cohort was conceived within the project "Socioeconomic differences in mortality" (IF IST 2646), as part of the National Statistical Program (PSN), approved by the Italian Data Protection Authority. The database developed by the Italian National Institute of Statistics (Istat) for this project permitted studying the differences in life expectancy and mortality (total and by cause) according to different demographic and socioeconomic characteristics observed in the 15^th^ Census of Population and Housing (2011)^28^.

The individuals recorded in the 2011 Census and resident in Italy were followed up from 2012 to 2018, until death, emigration, or end of the follow-up, whichever came first, yielding a maximum of 7 years of follow-up. Information on mortality was retrieved from the Causes of Death Register, which annually collects all deaths occurring in Italy, while the Resident Population Register was used to identify any exit from the cohort for emigration. A record linkage, using the fiscal code (a unique personal identification number issued to all residents in Italy at birth or upon request) as linkage key, connected all the archives. The reliability of the fiscal code was very high in all the registers, making it possible to link 97.1% of all deaths among the Census population occurring in Italy in the period 2012–2014^29^. Since there is no reason to believe that the reliability of the fiscal code reported in all registers decreased over the subsequent years, the performance of the record linkage was expected to be equally high.

Istat released a fully anonymised database for this study containing the number of deaths and person-years for the population strata defined through all the variables that were used for the analyses.

### Exposure

We considered as exposure immigration status, measured through citizenship, dichotomized in Italian and foreign. All the residents in Italy without Italian citizenship (including stateless) were considered as immigrants. In Italy, immigrants can obtain citizenship by marriage or by application after a period ranging between 3 and 10 consecutive years of legal residence. Moreover, children born in Italy to foreign parents can obtain citizenship after their 18th birthday. For all of these reasons, we considered citizenship the best proxy of immigrant status in Italy, rather than country of birth, at least to assess the most recent immigrations^2,30^.

### Outcome

The present study considered as the main outcome the deaths occurring between 2012 and 2018 in the study population. Overall mortality was analyzed, with a focus on specific causes. The causes of death were classified according to the 10^th^ revision of the International Classification of Diseases and Related Health Problems (ICD-10). Istat has adopted ICD-10 since 2003. Following updates were taken into account in the selection of the causes of death considered for the present analysis. The complete list of causes considered with ICD-10 codes is provided in the “Appendix”^2^.

### Other factors

We considered as potential confounders of the association between citizenship and mortality other demographic and socioeconomic characteristics of the population recorded in the Census: age at death, sex, area of residence in Italy, and area of origin.

Age at death was categorized in six classes (< 15, 15–29, 30–44, 45–64, 65–74, 75 + years old).

The geographic area of residence was categorized on the basis of the classification in five broad areas (macro areas) defined by Istat: North-West (Piedmont, Valle d’Aosta, Lombardy, Liguria), North-East (Trentino-Alto Adige, Veneto, Friuli-Venetia Giulia, Emilia-Romagna), Centre (Tuscany, Umbria, Marche, Latium), South (Abruzzo, Molise, Campania, Apulia, Basilicata, Calabria), and Islands (Sicily and Sardinia).

Countries of origin were categorized as follows, according to the ISTAT classification^31^, that was partially modified grouping some macro areas: European Union (before 2004), Central-Eastern Europe, other European countries, North Africa, Sub-Saharan Africa, North America, Central-South America, Central-West Asia, East Asia, Oceania, stateless.

### Statistical analysis

Baseline sociodemographic characteristics of the cohorts (person years, deaths) were described separately for Italians and immigrants, and the *p*-value of the differences in the proportion of deaths between the two groups were calculated using Chi-square method.

Crude mortality rates per 100,000 person years were calculated by citizenship, sex, area of origin, and cause of death.

To investigate mortality patterns by area of origin and by cause of death, given the small numbers in some mortality strata, age-standardized mortality ratios (SMRs) were computed using the Italian age-specific mortality rates as standard, and 95% confidence intervals (95% CI) were calculated^32^.

To evaluate the effect of citizenship on mortality, a multivariable quasi-Poisson regression model for overdispersed count data with log link function^33^, stratified by sex, was performed, taking into account age at death, and area of residence.

All analyses were performed using SAS® System version 9.3 (2^nd^ release SAS 9.3 TS1M2).

### Ethics approval

The cohort was conceived within the project "Socioeconomic differences in mortality" (IF IST 2646), as part of the National Statistical Program (PSN), approved by the Italian Data Protection Authority.

## Results

The cohort of Italians and immigrants enrolled in Census 2011 and followed up to 2018 included 59,227,313 subjects, accounting for 398,499,648 person years. Immigrants accounted for 7% of the subjects and for 0.6% of the 4,103,769 deaths observed during follow-up.

**Figure 1:**
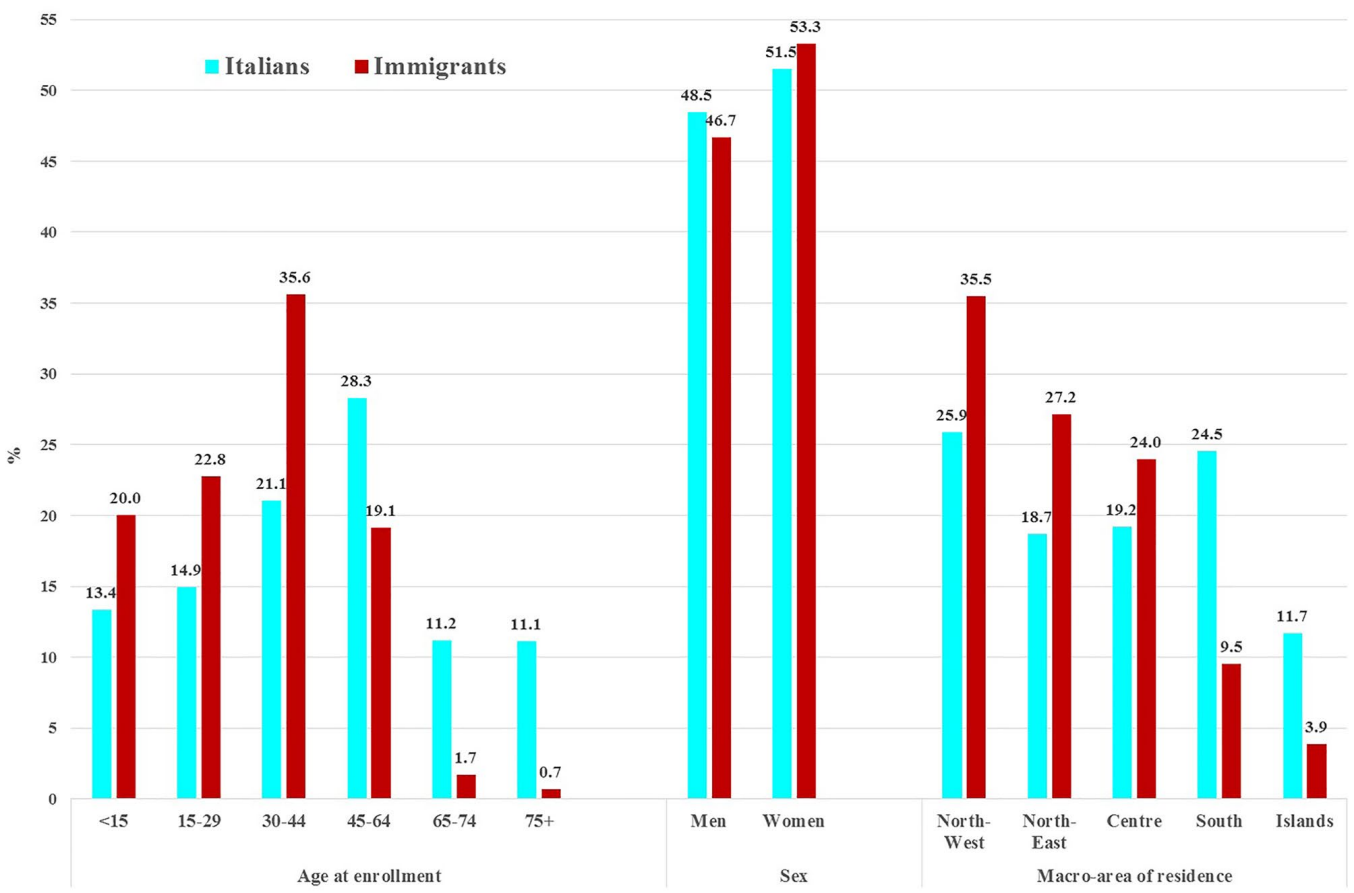
Differences between Italians and immigrants in the distribution of the sociodemographic characteristics.

We observed significant (*p* < 0.001) differences between Italians and immigrants in the distribution of the sociodemographic characteristics (Fig. 1). Compared to Italians, immigrants had a higher proportion of subjects age < 45 years old (78.5% vs 49.4%). Among immigrants the proportion of women (53.3 vs 51.5%) and of residents in Northern Italy (62.6% vs 44.6%) was higher than that of Italians.

About 50% of all immigrants came from Central-Eastern Europe, followed by North Africa (14.4%) and East Asia (8.5%) (data not shown).

The crude rates showed an excess in mortality for Italians compared to immigrants; in particular, the excess was about tenfold for males and 14-fold for females. When observing the standardized mortality ratios (SMR), we found that mortality among immigrants was half that among Italians, both for men (SMR: 0.52) and for women (SMR: 0.51). Moreover, the SMRs described a lower mortality of immigrants from all macro areas of origin, except for stateless persons, with the lowest SMRs observed for those coming from areas outside Europe, in particular North Africa and Oceania (Table 1).

**Table 1:** Standardized mortality ratios (SMR) with 95% CI, by citizenship and area of origin, stratified by sex. Istat, 2012–18.

Sex	Citizenship	Deaths	Crude mortality rate *100,000 person-years	SMR	Lower 95% CI	Upper 95% CI
Males	Italians	1,954,605	1,086.8	1	–	–
	Immigrants	13,526	105.7	0.52	0.51	0.54
Females	Italians	2,123,874	1110.7	1	–	–
	Immigrants	11,764	80.4	0.51	0.50	0.52
**Area of origin**						
Males	Italy	1,954,605	1086.8	1	–	–
	European Union (before 2004)	1810	577.2	0.55	0.52	0.58
	Central-eastern Europe	5719	99.5	0.56	0.54	0.57
	Other European countries	471	1584.5	0.78	0.71	0.86
	Northern Africa	1686	75.3	0.39	0.38	0.41
	Subsaharan Africa	966	91.7	0.61	0.57	0.65
	Northern America	291	753.4	0.51	0.46	0.58
	Central-southern America	548	69.8	0.39	0.35	0.42
	Central-western Asia	1191	79.4	0.56	0.53	0.60
	Eastern Asia	812	74.7	0.45	0.42	0.49
	Oceania	20	369.5	0.23	0.14	0.35
	Stateless	12	894.1	1.48	0.76	2.58
Females	Italy	2,123,874	1110.7	1	–	–
	European Union (before 2004)	2088	390.2	0.69	0.66	0.72
	Central-eastern Europe	5660	71.8	0.50	0.49	0.51
	Other European countries	374	920.2	0.79	0.71	0.87
	Northern Africa	781	45.5	0.31	0.29	0.34
	Subsaharan Africa	581	75.4	0.79	0.72	0.85
	Northern America	166	329.6	0.52	0.44	0.60
	Central-southern America	918	67.5	0.45	0.42	0.48
	Central-western Asia	480	47.7	0.45	0.41	0.49
	Eastern Asia	692	55.0	0.47	0.44	0.51
	Oceania	14	172.1	0.23	0.13	0.39
	Stateless	10	773.7	2.06	0.99	3.79

The multivariable quasi-Poisson regression analysis confirmed, after adjusting for age and macro area of residence in Italy, a lower all-cause mortality for immigrants compared to Italians, both among men (RR: 0.46; 95% CI 0.34–0.63) and women (RR: 0.44; 95% CI 0.33–0.57). No significant difference by macro area of residence in Italy was observed (data not shown).

The SMRs by cause of death showed that in some cases, mortality was higher in immigrants than in Italians, especially among women. Among males (Table 2) tuberculosis was the only cause with an excess in mortality among immigrant men (SMR 4.58); among immigrant women (Table 3), significantly higher mortality compared to the Italians was observed for tuberculosis (SMR 4.73), cervical cancer (SMR 1.58), for complications of pregnancy, childbirth, and puerperium (SMR 1.36), and for homicide (SMR 2.13).

**Table 2:** Number of deaths, crude mortality rate * 10,000 person-years and standardized mortality ratios (SMR with 95% CI) of immigrants vs Italians, by cause of death and citizenship. Istat, 2012–18—Males.

Cause of death	Italians (PY = 179,851,785)		Immigrants (PY = 12,800,677)		SMR	Lower 95% CI	Upper 95% CI
	Deaths	Crude mortality Rate *100,000	Deaths	Crude mortality Rate *100,000			
Infectious and parasitic diseases	40,782	22.68	406	3.17	0.62	0.56	0.68
Infection related*	151,025	83.97	1286	10.05	0.60	0.57	0.63
Tuberculosis (TB)	803	0.45	46	0.36	4.58	3.35	6.10
**Neoplasms**	657,500	365.58	4635	36.21	0.49	0.47	0.50
Upper aero-digestive tract (UADT)	31,359	17.44	276	2.16	0.46	0.41	0.51
Lung + trachea	159,621	88.75	1153	9.01	0.52	0.49	0.55
Colon rectum, recto-sigmoid junction, and anus	68,843	38.28	376	2.94	0.39	0.35	0.43
Stomach	36,854	20.49	337	2.63	0.61	0.55	0.68
Liver, gallbladder, other and unspecified parts of biliary tract	51,813	28.81	408	3.19	0.52	0.47	0.57
Non-Hodgkin lymphoma	17,183	9.55	159	1.24	0.55	0.47	0.65
Leukaemia	22,518	12.52	195	1.52	0.54	0.47	0.62
Endocrine, nutritional and metabolic diseases, and immunity disorders	80,398	44.70	429	3.35	0.43	0.39	0.47
Diseases of the blood and blood-forming organs	7149	3.97	36	0.28	0.42	0.30	0.58
Mental disorders	44,879	24.95	160	1.25	0.39	0.33	0.45
Diseases of the nervous system and sense organs	76,680	42.64	399	3.12	0.43	0.38	0.47
Diseases of the circulatory system	647,223	359.86	3511	27.43	0.52	0.51	0.54
Ischemic heart diseases	234,510	130.39	1354	10.58	0.52	0.49	0.54
Cerebrovascular diseases	152,935	85.03	797	6.23	0.60	0.56	0.64
Diseases of the respiratory system	164,400	91.41	642	5.02	0.47	0.44	0.51
Diseases of the digestive system	72,965	40.57	586	4.58	0.50	0.46	0.54
Diseases of the genitourinary system	36,970	20.56	142	1.11	0.47	0.40	0.55
Diseases of the skin and subcutaneous tissue	2546	1.42	7	0.05	0.28	0.11	0.58
Diseases of the musculoskeletal system and connective tissue	6513	3.62	53	0.41	0.61	0.46	0.80
Congenital anomalies	3151	1.75	49	0.38	0.33	0.24	0.43
Certain conditions originating in the perinatal period	151	0.08	6	0.05	0.51	0.19	1.10
Symptoms, signs, and ill-defined conditions	29,262	16.27	312	2.44	0.53	0.48	0.60
External causes of injury and poisoning	84,036	46.73	2153	16.82	0.73	0.70	0.76
Suicide	19,309	10.74	526	4.11	0.52	0.48	0.56
Homicide	1197	0.67	85	0.66	0.99	0.79	1.23

*Infection related tumours, infection related heart diseases, hepatitis, pneumonia, septicemia, infection related nervous system disease, AIDS.

**Table 3:** Number of deaths, crude mortality rate * 100,000 person-years and standardized mortality ratios (SMR with 95% CI) of immigrants vs Italians, by cause of death and citizenship. Istat, 2012–18—Females.

Cause of death	Italians (PY = 191,220,267)		Immigrants (PY = 14,626,920)		SMR	Lower 95% CI	Upper 95% CI
	Deaths	Crude rate *100,000	Deaths	Crude rate *100,000			
Infectious and parasitic diseases	47,185	24.68	331	2.26	0.66	0.59	0.74
Infection related*	133,395	69.76	1104	7.55	0.68	0.65	0.73
Tuberculosis (TB)	549	0.29	30	0.21	4.72	3.19	6.75
**Neoplasms**	518,667	271.24	5104	34.89	0.50	0.48	0.51
Upper aero-digestive tract (UADT)	10,608	5.55	126	0.86	0.57	0.48	0.68
Lung + trachea	62,050	32.45	633	4.33	0.46	0.42	0.50
Colon rectum, recto-sigmoid junction, and anus	57,957	30.31	434	2.97	0.46	0.42	0.51
Stomach	25,976	13.58	278	1.90	0.63	0.56	0.71
Liver, gallbladder, other and unspecified parts of biliary tract	34,081	17.82	239	1.63	0.49	0.43	0.56
Breast	81,446	42.59	985	6.73	0.44	0.42	0.47
Cervix uteri	2745	1.44	190	1.30	1.58	1.37	1.83
Non-Hodgkin lymphoma	14,343	7.50	135	0.92	0.53	0.45	0.63
Leukaemia	17,830	9.32	185	1.26	0.57	0.49	0.66
Endocrine, nutritional and metabolic diseases, and immunity disorders	104,122	54.45	364	2.49	0.38	0.34	0.42
Diseases of the blood and blood-forming organs	11,296	5.91	64	0.44	0.60	0.46	0.76
Mental disorders	91,260	47.73	210	1.44	0.42	0.37	0.48
Diseases of the nervous system and sense organs	102,048	53.37	395	2.70	0.39	0.35	0.43
Diseases of the circulatory system	845,882	442.36	2849	19.48	0.51	0.49	0.52
Ischemic heart diseases	222,404	116.31	698	4.77	0.46	0.43	0.50
Cerebrovascular diseases	234,191	122.47	909	6.21	0.58	0.55	0.62
Diseases of the respiratory system	143,715	75.16	530	3.62	0.51	0.47	0.56
Diseases of the digestive system	76,758	40.14	514	3.51	0.62	0.56	0.67
Diseases of the genitourinary system	42,448	22.20	129	0.88	0.43	0.36	0.51
Complications of pregnancy, childbirth, and the puerperium	53	0.03	10	0.07	1.36	0.65	2.51
Diseases of the skin and subcutaneous tissue	5436	2.84	23	0.16	0.61	0.39	0.91
Diseases of the musculoskeletal system and connective tissue	15,394	8.05	96	0.66	0.56	0.45	0.68
Congenital anomalies	2927	1.53	41	0.28	0.28	0.20	0.38
Certain conditions originating in the perinatal period	107	0.06	2	0.01	0.21	0.02	0.78
Symptoms, signs, and ill-defined conditions	51,435	26.90	235	1.61	0.60	0.53	0.69
External causes of injury and poisoning	65,141	34.07	867	5.93	0.80	0.75	0.86
Suicide	5447	2.85	267	1.83	0.80	0.70	0.90
Homicide	622	0.33	78	0.53	2.13	1.68	2.66

*Infection related tumours, infection related heart diseases, hepatitis, pneumonia, septicemia, infection related nervous system disease, AIDS.

## Discussion

Our study found that immigrants resident in Italy had lower all-cause mortality than did Italians, a result that confirms previous results in Italy^2^ and in other countries^24,25,34,35^. The mortality advantage of immigrants was more pronounced for those from areas outside Europe, in particular from North Africa and Oceania. In contrast, a higher cause-specific mortality was observed for tuberculosis among immigrants of both sexes and, in immigrant women, for cervical cancer, for causes related to pregnancy, childbirth and puerperium, and for homicide.

The advantage in mortality over Italians may be explained by the “healthy migrant effect” hypothesis, based on the selection for migration of individuals that are healthier than the native people both of the country of origin and of the country of destination^1,8–10^. This effect is evident in particular in the first generations of immigrants^2^.

Another explanation is the so-called salmon bias effect, the selective remigration of a subsample of unhealthy immigrants to their country of origin when they expect to die shortly. This may result in an artificial underestimation of immigrant mortality rates because deaths occurring in their country of origin are not registered in the mortality statistics of the country of residence, without an official residence change, resulting in these foreign citizens becoming “statistically immortal”^1,2,8,10–14^.

A recent study conducted in Italy found that, when considering the deaths occurring in the country of origin, this increased the overall mortality rates of immigrants resident in Italy by 18.1% and the age-standardized mortality rate by 23.7%, even if the hypothesized salmon bias effect was not enough to explain the large difference in mortality rates between the two groups of population^17^.

The lower mortality observed among immigrants concerned all areas of origin, a finding that does not confirm the mortality excess among Sub-Saharan African people observed by a study conducted in the cities of Turin and Reggio Emilia^2^ and the findings of a study conducted in six European countries, showing a higher all-cause mortality in people from North Africa and Eastern Europe and in women from Sub-Saharan Africa^26^. However, we observed some differences in mortality between the immigrant areas of origin. In particular, mortality rates were higher in subjects coming from Europe and North America, perhaps due to the greater similarity of their sociodemographic characteristics with the Italian population.

The results of our study highlight that immigrants lose their advantage over Italians for some specific causes of death: tuberculosis and, only among women, for cervical cancer, for complications of pregnancy, childbirth, and puerperium, and for homicide.

The increased mortality for tuberculosis among both male and female immigrants could represent the attenuation of the healthy migrant effect due to more disadvantaged living conditions, inducing a reactivation of latent tuberculosis infection^36^; confirmation of this seems to be the finding of very few cases of active tuberculosis at the moment of arrival in the host country^37^. Previous studies reported higher occurrence of tuberculosis among immigrants^38,39^, suggesting that immigrants arriving in Italy are protected from the most severe consequences of the acute infections acquired in the host country (healthy migrant effect), but are exposed to a higher prevalence of some infections in their country of origin which cause diseases later in life^2^.

The increased mortality among immigrants for cervical cancer can be explained by the higher prevalence in many of the countries of origin of human papilloma virus (HPV)^40^, the main cause of cervical cancer. An additional explanation could be lower screening attendance and suboptimal access to cancer treatment^2,41,42^.

Among women, the mortality excess among immigrants compared to Italians for complications of pregnancy, childbirth, and puerperium is consistent with the results of a large meta-analysis that observed a doubled risk of dying during or after pregnancy for immigrant women in Western European countries when compared with native-born women^43^. Our findings seem to confirm that maternal and child health represents the main criticality in providing assistance to the foreign population in Italy^41,44–46^.

Moreover, the higher mortality for homicide in immigrant females than in Italian females could be explained by the greater social vulnerability, given factors that are more prevalent in immigrant communities, including social isolation, cultural attitudes, gender roles, and fewer employment options^2,24–26,47^.

We did not find any excess mortality from stomach cancer, contrary to the findings of two systematic reviews^48,49^ as well as almost all studies. This may be surprising, since stomach cancer is strongly linked with socioeconomic status, and immigrants generally have a lower socioeconomic level than do Italians^50^. However, Pabla’s extensive systematic review, covering about 40 years, found no Italian or Italian-referenced publication related to stomach cancer among immigrants^48^. A hypothesis to explain this difference could be the different demographic composition of immigrants in Italy, with a prevalence of communities of European origin, which are less prone to cancers, such as stomach cancer, that are related to infections experienced in early life^49^.

## Strengths and limitations

The study has considerable strengths. First, this is the first study to analyze mortality among immigrants covering the entire resident population in Italy. Its longitudinal approach made it possible to avoid any bias due to the numerator-denominator mismatch in ecological cross-sectional studies and to calculate the exact person-time at risk through a link between individual population records and death registries. Second, to our knowledge, this is the first mortality study based on the whole population of residents in Italy, making it possible to provide a detailed picture of the phenomenon.

However, our study suffers from some limitations. First, like other studies dealing with mortality among immigrants, our study presents the potential bias of unregistered remigration and deaths of immigrants, which may have determined potential overcoverage and salmon bias^1,2,8–18^. Second, since our study was based on registers of the resident population, immigrants who were undocumented, a particularly vulnerable subpopulation, were not included. However, the percentage of undocumented immigrants in Italy is estimated to be only 0.5% of the total resident population^51^.

In addition, as shown by Gimeno-Feliu et al.^52^, the use of citizenship instead of country of birth to define immigrant status, as in our study, can lead to an underestimation of mortality among foreigners compared to natives. This is especially true for individuals who acquired citizenship after a long stay in the country, who are expected to have mortality risks closer to those of natives. Unfortunately, we do not have information in our database on either country of birth or length of stay in Italy.

## Conclusions

Although immigration to Italy is no longer a recent phenomenon, and the presence of immigrants is acquiring structural characteristics, our study confirms their health advantage, with lower mortality than that of Italians from almost every cause. This lower mortality in the immigrant population is also observed for all areas of origin. These findings partially differ from a previous Italian longitudinal study and a European study^2,26^. Future studies could be designed with a nationwide open cohort approach, which is particularly useful for studying an extremely dynamic population such as the immigrant population, taking into account further sources to reduce the potential effects of salmon bias.

## Supplementary Information

Supplementary Information.

## Data Availability

Statistical analysis were carried out within a research protocol between Istat and INMP, both national public Institute. The database used for the analysis are subject to the legal restrictions established by the European privacy law. The database that support the findings of this study were made available by Istat, but restriction apply to the availability of these data, which were used under license for the current study, and are thus not publicly available. Specific statistical analysis can be requested and agreed with Istat.
