# Supplementary material for: Nationwide longitudinal population-based study on mortality in Italy by immigrant status
Source: Sci Rep. 2022 Jun 29;12:10986. doi: 10.1038/s41598-022-15290-8 (PMC9243023; doi:10.1038/s41598-022-15290-8)
Supplement: Supplementary file 1 — Supplementary Information. [file 41598_2022_15290_MOESM1_ESM.docx]

**Appendix**

**Table A1. Cause of deaths and ICD10 codes.**

| **Cause** | **ICD 10 codes** |
| --- | --- |
| infectious and parasitic diseases | A00–B99 |
| incfectious+related (Infection-related tumour, infection-related heart diseases, hepatitis, pneumonia, septicaemia, infection-related nervous system diseases, AIDS) | see Appendix, Table 2 |
| tuberculosis (TB) | A15-A19 |
| neoplasms | C00–D48 |
| upper aero-digestive tract (UADT) | C00-C14 |
| lung | C34 |
| colon rectum, rectosigmoid junction, and anus | C18-C21 |
| stomach | C16 |
| liver | C22 |
| breast | C50 |
| cervix uteri | C53 |
| non-Hodgkin lymphoma | C82-C83 |
| leukaemia | C91-C95 |
| endocrine, nutritional and metabolic diseases, and immunity disorders | E00-E90 + D80-D89 |
| diseases of the blood and blood-forming organs | D50-D77 |
| mental disorders | F00-F99 |
| diseases of the nervous system and sense organs | G00–G99 |
| diseases of the circulatory system | I00-I99 |
| ischemic heart diseases | I20-I25 |
| cerebrovascular diseases | I60-I69 |
| diseases of the respiratory system | J00-J99 |
| diseases of the digestive system | K00-K93 |
| diseases of the genitourinary system | N00-N99 |
| complications of pregnancy, childbirth, and the puerperiumc | O00-O9979 |
| diseases of the skin and subcutaneous tissue | L00-L99 |
| diseases of the musculoskeletal system and connective tissue | M00-M99 |
| congenital anomalies | Q00-Q99 |
| certain conditions originating in the perinatal period | P00-P96 |
| symptoms, signs, and ill-defined conditions | R00-R99 |
| injury and poisoning | S00–T98 |
| suicide | X60-X84 |
| homicide | X85-Y09 |

**Table A2. List of International Classification of Diseases 10th versions codes that were considered in the infection-related-death analysis**

| **Description** | **ICD-10 Codes** |
| --- | --- |
| **Acquired Immunodeficiency syndrome (AIDS)** | |
| HIV disease resulting in infectious and parasitic disease | B20 |
| HIV disease resulting in malignant neoplasms | B21 |
| HIV disease resulting in other specified diseases | B22 |
| HIV disease resulting in other conditions | B23 |
| unspecified HIV disease | B24 |
| **Tumour** | |
| malignant neoplasm of nasopharynx | C11 |
| non-cardia malignant neoplasm of stomach | C16.1-C16.9 |
| MALT-lymphoma | C88.4 |
| malignant neoplasm of liver and intrahepatic bile ducts | C22 |
| malignant neoplasm of anus, unspecified | C21.0 |
| malignant neoplasm of cervix uteri | C53 |
| Kaposi sarcoma | C46 |
| **Heart Disease** | |
| meningococcal heart disease | A39.5 |
| candidal endocarditis | B37.6 |
| acute rheumatic pericarditis | I01.0 |
| acute rheumatic endocarditis | I01.1 |
| acute rheumatic myocarditis | I01.2 |
| rheumatic myocarditis | I09.0 |
| chronic rheumatic pericarditis | I09.2 |
| infective pericarditis | I30.1 |
| other forms of acute pericarditis | I30.8 |
| acute and subacute infective endocarditis | I33.0 |
| acute endocarditis, unspecified | I33.9 |
| endocarditis, valve unspecified | I38 |
| infective myocarditis | I40.0 |
| isolated myocarditis | I40.1 |
| other acute myocarditis | I40.8 |
| acute myocarditis, unspecified | I40.9 |
| myocarditis in bacterial diseases classified elsewhere | I41.0 |
| myocarditis in viral diseases classified elsewhere | I41.1 |
| myocarditis in other infectious and parasitic diseases classified elsewhere | I41.2 |
| **Hepatitis** | |
| hepatitis A with hepatic coma | B15.0 |
| hepatitis A without hepatic coma | B15.9 |
| acute hepatitis B with delta-agent (coinfection) with hepatic coma | B16.0 |
| acute hepatitis B with delta-agent (coinfection) without hepatic coma | B16.1 |
| acute hepatitis B without delta-agent with hepatic coma | B16.2 |
| acute hepatitis B without delta-agent and without hepatic coma | B16.9 |
| acute delta-(super)infection of hepatitis B carrier | B17.0 |
| acute hepatitis C | B17.1 |
| acute hepatitis E | B17.2 |
| other specified acute viral hepatitis | B17.8 |
| chronic viral hepatitis B with delta-agent | B18.0 |
| chronic viral hepatitis B without delta-agent | B18.1 |
| chronic viral hepatitis C | B18.2 |
| other chronic viral hepatitis | B18.8 |
| chronic viral hepatitis, unspecified | B18.9 |
| unspecified viral hepatitis with coma | B19.0 |
| unspecified viral hepatitis without coma | B19.9 |
| **Pneumonia** | |
| tuberculosis of lung, confirmed by sputum microscopy with or without culture | A15.0 |
| tuberculosis of lung, confirmed by culture only | A15.1 |
| tuberculosis of lung, confirmed histologically | A15.2 |
| tuberculosis of lung, confirmed by unspecified means | A15.3 |
| tuberculosis of larynx, trachea and bronchus, confirmed bacteriologically and | A15.5 |
| histologically |  |
| tuberculosis of lung, bacteriologically and histologically negative | A16.0 |
| tuberculosis of lung, bacteriological and histological examination not done | A16.1 |
| tuberculosis of lung, without mention of bacteriological or histological confirmation | A16.2 |
| tuberculosis of larynx, trachea and bronchus, without mention of bacteriological or histological confirmation | A16.4 |
| pulmonary mycobacterial infection | A31.0 |
| pulmonary actinomycosis | A42.0 |
| pulmonary nocardiosis | A43.0 |
| legionnaires disease | A48.1 |
| varicella pneumonia | B01.2 |
| measles complicated by pneumonia ( | B05.2 |
| HIV disease resulting in Pneumocystis jirovecii pneumonia | B20.6 |
| HIV disease resulting in lymphoid interstitial pneumonitis | B22.1 |
| cytomegalovirus pneumonia | B25.0 |
| pulmonary candidiasis | B37.1 |
| acute pulmonary coccidioidomycosis | B38.0 |
| chronic pulmonary coccidioidomycosis | B38.1 |
| pulmonary coccidioidomycosis, unspecified | B38.2 |
| acute pulmonary histoplasmosis capsulati | B39.0 |
| chronic pulmonary histoplasmosis capsulati | B39.1 |
| pulmonary histoplasmosis capsulati, unspecified | B39.2 |
| acute pulmonary blastomycosis | B40.0 |
| chronic pulmonary blastomycosis | B40.1 |
| pulmonary blastomycosis, unspecified | B40.2 |
| pulmonary paracoccidioidomycosis | B41.0 |
| pulmonary sporotrichosis | B42.0 |
| Invasive pulmonary aspergillosis | B44.0 |
| other pulmonary aspergillosis | B44.1 |
| pulmonary cryptococcosis | B45.0 |
| pulmonary mucormycosis | B46.0 |
| pulmonary toxoplasmosis | B58.3 |
| echinococcus granulosus infection of lung | B67.1 |
| influenza with pneumonia, seasonal influenza virus identified | J10.0 |
| influenza with pneumonia, virus not identified | J11.0 |
| adenoviral pneumonia | J12.0 |
| respiratory syncytial virus pneumonia | J12.1 |
| parainfluenza virus pneumonia | J12.2 |
| other viral pneumonia | J12.8 |
| viral pneumonia, unspecified | J12.9 |
| pneumonia due to Streptococcus pneumoniae | J13 |
| pneumonia due to Haemophilus influenzae | J14 |
| pneumonia due to Klebsiella pneumoniae | J15.0 |
| pneumonia due to Pseudomonas | J15.1 |
| pneumonia due to staphylococcus | J15.2 |
| pneumonia due to streptococcus, group B | J15.3 |
| pneumonia due to other streptococci | J15.4 |
| pneumonia due to Escherichia coli | J15.5 |
| pneumonia due to other aerobic Gram-negative bacteria | J15.6 |
| pneumonia due to Mycoplasma pneumoniae | J15.7 |
| other bacterial pneumonia | J15.8 |
| bacterial pneumonia, unspecified | J15.9 |
| chlamydial pneumonia | J16.0 |
| pneumonia due to other specified infectious organisms | J16.8 |
| pneumonia in bacterial diseases classified elsewhere | J17.0 |
| pneumonia in viral diseases classified elsewhere | J17.1 |
| pneumonia in mycoses | J17.2 |
| pneumonia in parasitic diseases | J17.3 |
| pneumonia in other diseases classified elsewhere | J17.8 |
| bronchopneumonia unspecified | J18.0 |
| lobar pneumonia unspecified | J18.1 |
| other pneumonia, organisms unspecified | J18.8 |
| pneumonia unspecified | J18.9 |
| **Septicemia** | |
| sepsis due to streptococcus, group A | A40.0 |
| sepsis due to streptococcus, group B | A40.1 |
| sepsis due to streptococcus, group D | A40.2 |
| sepsis due to Streptococcus pneumoniae | A40.3 |
| other streptococcal sepsis | A40.8 |
| streptococcal sepsis unspecified | A40.9 |
| sepsis due to Staphylococcus aureus | A41.0 |
| sepsis due to other specified staphylococcus | A41.1 |
| sepsis due to unspecified staphylococcus | A41.2 |
| sepsis due to Haemophilus influenzae | A41.3 |
| sepsis due to anaerobes | A41.4 |
| sepsis due to other Gram-negative organisms | A41.5 |
| other specified sepsis | A41.8 |
| sepsis, unspecified | A41.9 |
| actinomycotic sepsis | A42.7 |
| **Nervous System** | |
| tuberculous meningitis | A17.0 |
| meningococcal meningitis | A39.0 |
| Waterhouse-Friderichsen syndrome | A39.1 |
| acute meningococcemia | A39.2 |
| chronic meningococcemia | A39.3 |
| meningococcemia unspecified | A39.4 |
| other meningococcal infections | A39.8 |
| meningococcal infection, unspecified | A39.9 |
| Japanese encephalitis | A83.0 |
| western equine encephalitis | A83.1 |
| eastern equine encephalitis | A83.2 |
| St Louis encephalitis | A83.3 |
| Australian encephalitis | A83.4 |
| California encephalitis | A83.5 |
| Rocio virus disease | A83.6 |
| other mosquito-borne viral encephalitis | A83.8 |
| mosquito-borne viral encephalitis, unspecified | A83.9 |
| far eastern tick-borne encephalitis (Russian spring-summer encephalitis) | A84.0 |
| central European tick-borne encephalitis | A84.1 |
| other tick-borne viral encephalitis | A84.8 |
| tick-borne viral encephalitis, unspecified | A84.9 |
| enterovirus encephalitis | A85.0 |
| adenoviral encephalitis | A85.1 |
| arthropod-borne viral encephalitis, unspecified | A85.2 |
| other specified viral encephalitis | A85.8 |
| unspecified viral encephalitis | A86 |
| enterovirus meningitis | A87.0 |
| adenoviral meningitis | A87.1 |
| lymphocytic choriomeningitis | A87.2 |
| other viral meningitis | A87.8 |
| viral meningitis, unspecified | A87.9 |
| other specified viral infections of central nervous system | A88.8 |
| unspecified viral infection of central nervous system | A89 |
| herpesviral meningitis | B00.3 |
| herpesviral encephalitis | B00.4 |
| varicella meningitis | B01.0 |
| varicella encephalitis | B01.1 |
| zoster encephalitis | B02.0 |
| zoster meningitis | B02.1 |
| measles complicated by encephalitis | B05.0 |
| measles complicated by meningitis | B05.1 |
| HIV disease resulting in Pneumocystis jirovecii pneumonia | B20.6 |
| HIV disease resulting in encephalopathy | B22.0 |
| HIV disease resulting in lymphoid interstitial pneumonitis | B22.1 |
| HIV disease resulting in wasting syndrome | B22.2 |
| cytomegalovirus pneumonia | B25.0 |
| mumps meningitis | B26.1 |
| mumps encephalitis | B26.2 |
| candida meningitis | B37.5 |
| candidal endocarditis | B37.6 |
| coccidioidomycosis meningitis | B38.4 |
| toxoplasma meningoencephalitis | B58.2 |
| haemophilus meningitis | G00.0 |
| pneumococcal meningitis | G00.1 |
| streptococcal meningitis | G00.2 |
| staphylococcal meningitis | G00.3 |
| other bacterial meningitis | G00.8 |
| bacterial meningitis, unspecified | G00.9 |
| meningitis in bacterial diseases classified elsewhere | G01 |
| meningitis in viral diseases classified elsewhere | G02.0 |
| meningitis mycoses | G02.1 |
| meningitis in other specified infectious and parasitic diseases classified elsewhere | G02.8 |
| nonpyogenic meningitis | G03.0 |
| chronic meningitis | G03.1 |
| benign recurrent meningitis (Mollaret) | G03.2 |
| meningitis due to other specified causes | G03.8 |
| meningitis unspecified | G03.9 |
| acute disseminated encephalitis | G04.0 |
| tropical spastic paraplegia | G04.1 |
| bacterial meningoencephalitis and meningomyelitis, not elsewhere classified | G04.2 |
| other encephalitis, myelitis and encephalomyelitis | G04.8 |
| encephalitis, myelitis and encephalomyelitis, unspecified | G04.9 |
| encephalitis, myelitis and encephalomyelitis in bacterial diseases classified elsewhere | G05.0 |
| encephalitis, myelitis and encephalomyelitis in viral diseases classified elsewhere | G05.1 |
| encephalitis, myelitis and encephalomyelitis in other infectious and parasitic diseases classified elsewhere | G05.2 |
| encephalitis, myelitis and encephalomyelitis in other diseases classified elsewhere | G05.8 |
| intracranial abscess and granuloma | G06.0 |
| intraspinal abscess and granuloma | G06.1 |
| extradural and subdural abscess, unspecified | G06.2 |
